# The impact of handling technique and handling frequency on laboratory mouse welfare is sex-specific

**DOI:** 10.1038/s41598-020-74279-3

**Published:** 2020-10-14

**Authors:** Federica Sensini, Dragos Inta, Rupert Palme, Christiane Brandwein, Natascha Pfeiffer, Marco Andrea Riva, Peter Gass, Anne Stephanie Mallien

**Affiliations:** 1 Department of Pharmacological and Biomolecular Sciences, University of Milan, Milan, Italy; 2 RG Animal Models in Psychiatry, Department of Psychiatry and Psychotherapy, Central Institute of Mental Health, Medical Faculty Mannheim, Heidelberg University, Mannheim, Germany; 3 Department of Psychiatry (UPK), University of Basel, Basel, Switzerland; 4 Department of Biomedical Sciences, University of Veterinary Medicine, Vienna, Austria

**Keywords:** Behavioural methods, Biological models, Biomarkers

## Abstract

Handling is a well-known source of stress to laboratory animals and can affect variability of results and even compromise animal welfare. The conventional tail handling in mice has been shown to induce aversion and anxiety-like behaviour. Recent findings demonstrate that the use of alternative handling techniques, e.g. tunnel handling, can mitigate negative handling-induced effects. Here, we show that technique and frequency of handling influence affective behaviour and stress hormone release of subjects in a sex-dependent manner. While frequent tail handling led to a reduction of wellbeing-associated burrowing and increased despair-like behaviour in male mice, females seemed unaffected. Instead, they displayed a stress response to a low handling frequency, which was not detectable in males. This could suggest that in terms of refinement, the impact in handling could differ between the sexes. Independently from this observation, both sexes preferred to interact with the tunnel. Mice generally explored the tunnel more often than the tail-handling hands of the experimenter and showed more positively rated approaches, e.g. touching or climbing, and at the same time, less defensive burrowing, indicating a strong preference for the tunnel.

## Introduction

Handling is described as the physical interaction between a laboratory animal and a human operator—be it during maintenance or scientific procedures. Different techniques of handling are practiced in laboratories all over the world. It is what researchers experience in their daily work life. And so do the subjects. The handling technique can influence the wellbeing of the animals and even the outcome of experiments, particularly those concerning spontaneous behaviours^1,2^. Recent research points out that the handling process is a major modulator of the welfare of laboratory mice^3–6^.

The conventional practice for handling mice is to lift them by taking the base of the tail between thumb and forefinger. However, this tail-handling technique has been questioned increasingly. Hurst and West^4^ were the first to show that tail handling induces anxiety-like behaviour and aversion to human contact. Further studies confirmed that tail handled mice avoid potential aversive situations, e.g. the open arms of the elevated plus-maze or in proximity of the hand of the experimenter^3–5,7,8^. This reduced urge to explore novel environments or situations can confound any tests based on explorative behaviour including light–dark paradigm, open field and hole-board test^9–13^. Despite the evidences that tail handling impairs the emotional state of mice and may confound the results of behavioural experiments^14,15^, this method is still predominant. It is often regarded as quick, easy to learn for new users and can be performed without cooperation of the animals^4^.

Alternative methods such as *handling tunnels* mitigate handling induced effects^4^ by eliminating the need for direct contact with the subject. The mouse is gently guided into a tunnel, which can then be lifted. Unlike tail handled mice, tunnel handled mice display an increase in voluntary interaction with the handler and a progressive habituation to the human contact^5^. Additionally, the mice show less anxiety and stress markers such as urination and defecation during human contact^4^. Therefore, whenever it is crucial to minimize anxiety in experimental mice, the tunnel method^5^ seems to be preferable over tail handling^5^. Apparently, non-aversive handling methods are a valuable tool to refine animal experiments, as requested by the 3Rs principles. Nevertheless, tunnel handling has rarely been implemented in the daily routine of laboratories^16^. The major criticism is on feasibility, since implementing non-aversive handling methods is often considered too time-costly: especially the initial habituation is thought to be labour-intensive and sometimes even stressful for the animal. Routines such as cage changes can be more time-consuming, particularly during the initial training to the handling technique^17^. Often, tunnel handling is also falsely associated with the necessity of a permanent tunnel as environmental enrichment in the home cage. Although environmental enrichment is generally endorsed to refine animal experiments, it might be advisable to limit enrichment under certain circumstances^18^. Hence, the experimenters are hesitant to use the tunnel merely as a handling device, although tunnel handling is equally well accepted by mice, also when the tunnel is solely used as a handling device without being a part of their home cage environment^5^.

Another important parameter for handling is habituation. While the frequent exposure to handling can lead to habituation effects^19^, a repetitive exposure to stress can induce depression-like symptoms^20,21^. We hypothesized that the frequently inflicted anxiogenic experience of regular tail handling might induce chronic stress with the result of despair behaviour—a depression-associated behaviour, as chronic exposure to stress depicts a commonly used model for depression in rodents^22^. The chronic exposure to various and often unpredictable mild stressors, however, would probably lead to much higher severity in the chronic mild stress models of depression. Yet, a recent study already demonstrated an anhedonia-like phenotype in tail handled mice—another hallmark of depression. In this, Clarkson et al. found that the hedonic consumption of a palatable sucrose solution was significantly decreased after tail handling^3^. We therefore decided to investigate here the impact of handling frequency on despair. The more frequent (daily) tail handling was expected to elicit a higher stress load and therefore induce a stronger response. We also analysed the stress hormone response via faecal corticosterone metabolites (FCMs) and voluntary burrowing as wellbeing-associated parameters^23–26^.

Additionally, we aimed to reproduce the effects of handling on voluntary interaction with the experimenter to display a potential preference of the subjects towards the tunnel—which would further reinforce the idea of tunnel handling as a suitable way to refine experiments with mice.

## Material and methods

### Animals and housing

All procedures were performed in the experimental unit of the animal facility of the CIMH. The subjects were 32 males and 32 females naïve C57BL/6NCrl mice (Charles River Laboratory, Sulzfeld, Germany; age: PND 56–62 at arrival, and PND 91–97 at the beginning of burrowing training). Before the onset of experiments, the animals were accustomed to a the housing room with 21–23 °C room temperature and 50–60% humidity and a reversed 12:12 dark–light cycle (lights on at 7 pm) for 2 weeks. They wereindividually housed in Macrolon type II (370 cm^2^, Tecniplast, Milan, Italy) cages with aspen wood bedding (ABEDD LTE E-002, ssniff-Spezialdiäten, Soest, Germany) and nesting material (cellulose tissue). They were supplied with food pellets (LasQCdiet Rod16-H, LasVendi GmbH, Soest, Germany) and tap water ad libitum. Behavioural experiments were conducted during the active phase of the animals (8–12 pm) unless further specified. All procedures were approved by the German animal welfare authorities (Regierungspräsidium Karlsruhe; 35-9185-81-G-146-13) and performed strictly according to the regulations of animal experimentation within the European Union (European Communities Council Directive 2010/63/EU).

Separated for sex, mice were randomly assigned to one handling procedure: tail or tunnel (for each sex n = 16). We further divided the groups into subgroups, which received different frequencies of handling (n = 8 for each sex): daily (from Monday to Friday) or once per week.

### Handling and interaction test

Before the onset of the respective handling, all mice were tail handled once a week during cage change. Mice were familiarized with the respective handling procedure during an adaptation period of 2 weeks, during which they were handled for routine maintenance and during the *interaction test* (see below). The procedure involved 30 s of handling, 60 s of rest in the home cage and repeated handling for 30 s. Tail handling was performed as follows: the experimenter gently grasped the tail and put the mouse on the sleeve of the laboratory coat for 30 s before returning it to the home cage. For tunnel handling, the mouse was guided into the tunnel (opaque, 130 mm length, 55 mm diameter) and then lifted for 30 s. The experimenter wore Nitrile Powder-free gloves (Abena Classic, Abena A/S, Aabenraa, Denmark) during all handling procedures.

Daily handled mice performed the *interaction test* for 3 days within 6 days (Monday, Wednesday and Friday), weekly handled only once (Wednesday) The procedure was the following: After the cage lid and nesting material were removed from the home cage, the gloved hand of the experimenter or the handling tunnel was introduced for 60 s. We assessed the voluntary interaction towards the carrier (hand or tunnel) and counted the number of approaches. Subsequently, each mouse was handled for 30 s by the assigned method, followed by 60 s of rest in the home cage without hand or tunnel. Then the interaction procedure was repeated, and the voluntary interaction was assessed again.

We counted sniffing, touching, climbing on the tunnel or the handler’s hand and burrowing as parameters of voluntary interaction. The sniffing behaviour was rated as a neutral exploratory behaviour while touching and climbing behaviours were considered as a more positive interaction, exhibiting the willingness to interact or even socialize. In this constellation, we also evaluate burrowing, as a defensive and negative interaction, similar to the behaviour displayed in the marble burying test, where burrowing is a validated indicator for an anxiety-like response. In fact, while burrowing is considered an indicator of wellbeing in normal circumstances, aversive stimuli can generate defensive burying behaviour towards novel potentially threatening objects^27,28^.

### Burrowing

Burrowing is an innate behaviour in rodents that is frequently used to assess the overall wellbeing of mice^29–31^. A plastic bottle (standard opaque water bottle, 250 ml, 150 mm length, 55 mm diameter) was filled with before mentioned food pellets and introduced into the home cage one hour before the onset of the dark phase. Mice were free to burrow the substrate out of the bottle^32^. The percentage of burrowed material was measured after 6 h. We familiarized the mice with the burrowing schedule for 5 days before the onset of the respective handling treatment. The burrowing performance was detected 2 days before and after the initial handling adaptation phase.

### Forced swim test

The Forced Swim Test measures depressive-like behaviour. Mice were transferred to an experimental room where they were placed into a glass cylinder (23 cm high, 13 cm in diameter) filled with water (22 °C) up to a height of 8 cm. Within 6 min the onset and the percentage of floating were determined as described elsewhere^33,34^.

### Fecal corticosterone metabolites (FCMs)

12 days after the start of the experimental handling technique, fecal samples for stress monitoring were collected from the cages 24 h after the cage change as previously described. This non-invasive method circumvents additional handling and restraint for blood sampling which could confound the experimental desgin. Fecal samples were extracted with methanol using a standard protocol^35,36^. Briefly, an aliquot (50 mg) of each well-homogenized, dried fecal sample was mixed with 1 ml 80% methanol, vortexed/shaken for 30 min and subsequently centrifuged for 10 min at 2500×*g*. The supernatant was then analysed using a 5α-pregnane-3β,11β,21-triol-20-one enzyme immunoassay as previously described and successfully validated for use in mice^37,38^.

### Statistics

Statistical analyses were carried out using IBM SPSS Statistics 24. Differences were considered significant at p < 0.05. The FST and handling interaction data were analysed using three-way ANOVA with the factors ‘handling technique’, ‘handling frequency’ and ‘sex’ and, when appropriate, using repeated-measures ANOVA. Mann–Whitney U-Tests for independent samples and Wilcoxon test for related samples were used to analyze burrowing behaviour, as well as FCM concentrations. We additionally analysed the development of interaction behavior over the course of the training using linear mixed models (LMM) with parameter ~ time*condition + (1|ID). ID was considered a random effect. The repeated covariance type was set to scaled identity. The intercept for the default model was 5.0 s (pre)6 and 6.19 s (post) seconds for sniffing, 3.00 s (pre) and 3.75 s (post) for touching, 1.53 s (pre) and 1.81 s (post) for climbing and 0.81 s (pre) and 1.75 (post) for burrowing. Post-hoc analyses were Šidák corrected.

No animals were excluded from the study of from any statistical analyses. The single animal served as an experimental unit.

## Results

### Body weight was not affected by handling technique

Mice showed increase of body weight over time in the weekly assessments (before, during and after interaction assessment: F(4,224) = 82.417, p < 0.001) and the typical sex difference (F(1,56) = 169.824, p < 0.001). Additionally, males gained weight more quickly (time*sex F(4,224) = 12.172, p < 0.001). We did not detect treatment related differences Another interaction was between sex and intensity, where males weight less when handled daily (sex*intensity: F(1,56) = 3.543, p = 0.065).

#### Male mice showed stronger impairments in affective behaviour after tail handling

**Figure 1:**
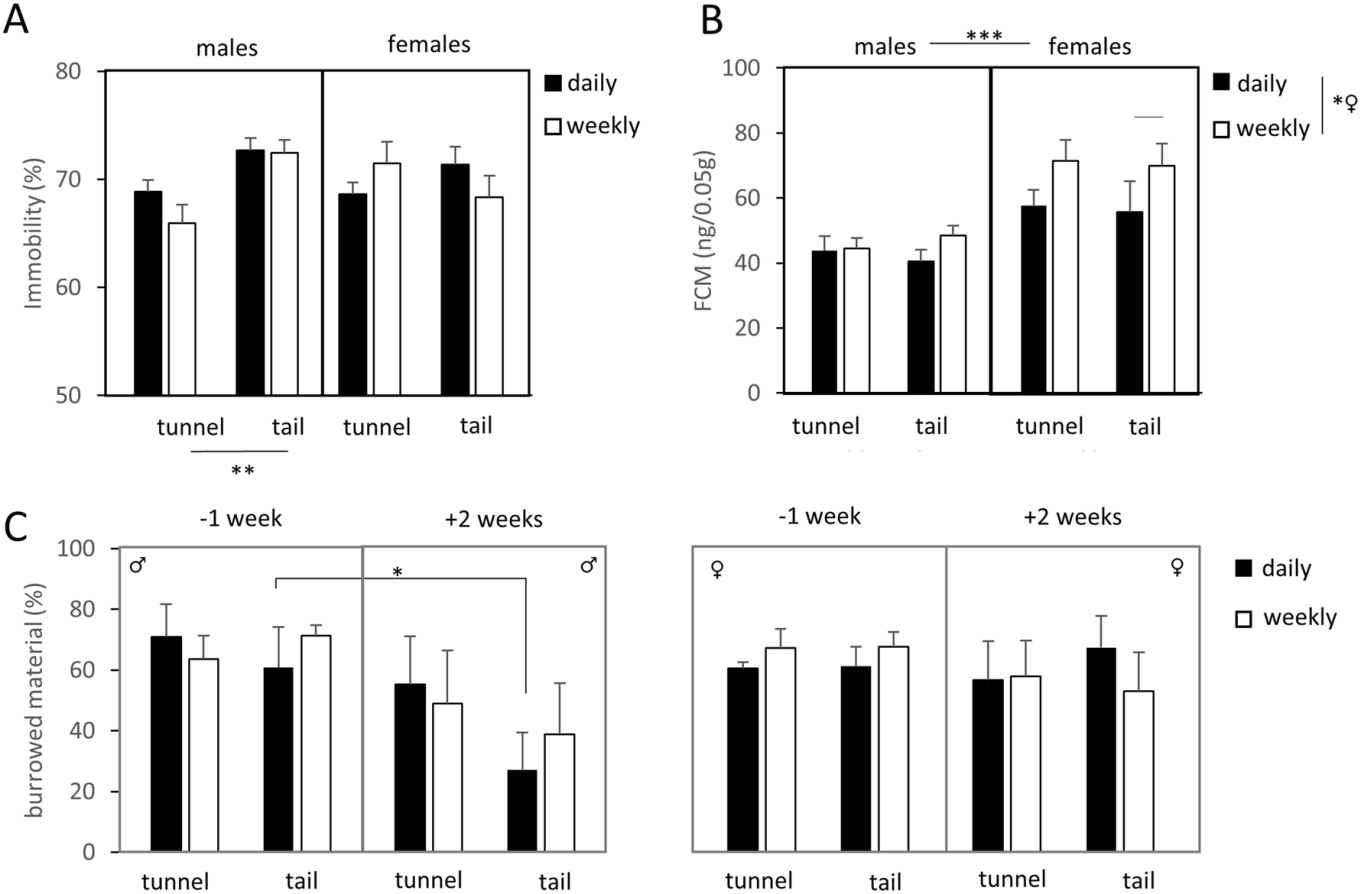
Different dimensions of handling influenced the assessed parameters: (**A**) handling style influenced the depression-associated behaviour in males; (**B**) handling frequency influenced the FCM concentration in females; (**C**) handling technique influenced the burrowing behaviour in males. Handling evoked alterations in (**A**) depressive-like behaviour, (**B**) FCM concentrations and (**C**) burrowing performance. Males showed a significant increase in immobility and a decrease of burrowing due to tail handling, which indicates an impaired wellbeing. These parameters were unaltered in females. However, the FCM concentration of males remained unchanged, while the stress hormone response was more sensitive to the intense daily handling procedure, regardless of the applied technique of handling. Data are represented as mean ± SEM. *p < 0.05, **p < 0.01, ***p < 0.001.

We detected the effects of the handling technique on depressive-like behaviour in the FST and reduced activity in the burrowing test (Fig. 1A,C).

The handling technique significantly influenced the immobility in the FST (technique: F_(1,56)_ = 4.458, p = 0.039) and, although no sex-effect was apparent, an interaction between sex and handling technique was revealed (technique*sex: F_(1,56)_ = 5.080, p = 0.028), where tail handling was linked to more immobile behaviour in males (males: technique F_(1,30)_ = 10.107, p = 0.003). Females showed no significant behavioural alterations. Handling frequency did not significantly influence immobility.

We did not observe differences linked to sex, technique or frequency of handling on burrowing behaviour. However, we did find a significant decrease in burrowing in males after daily tail handling for 2 weeks (Wilcoxon z = − 2.100 p = 0.036). The performance of females was not altered by any handling treatment.

#### Concentrations of fecal corticosterone metabolites (FCMs) were lower in more frequently handled mice

Higher concentrations of FCMs were found in female mice (sex U_(1,41)_ = 95.000, p < 0.001; Fig. 1B). Sex-dependent analysis revealed no differences due to handling frequency for males, but a significant increase due to weekly handling in females (females: frequency U_(1,22)_ = 34.000, p = 0.028). The handling technique did not influence FCM concentrations in both sexes.

#### Mice exhibit more positive interaction towards the tunnel

**Figure 2:**
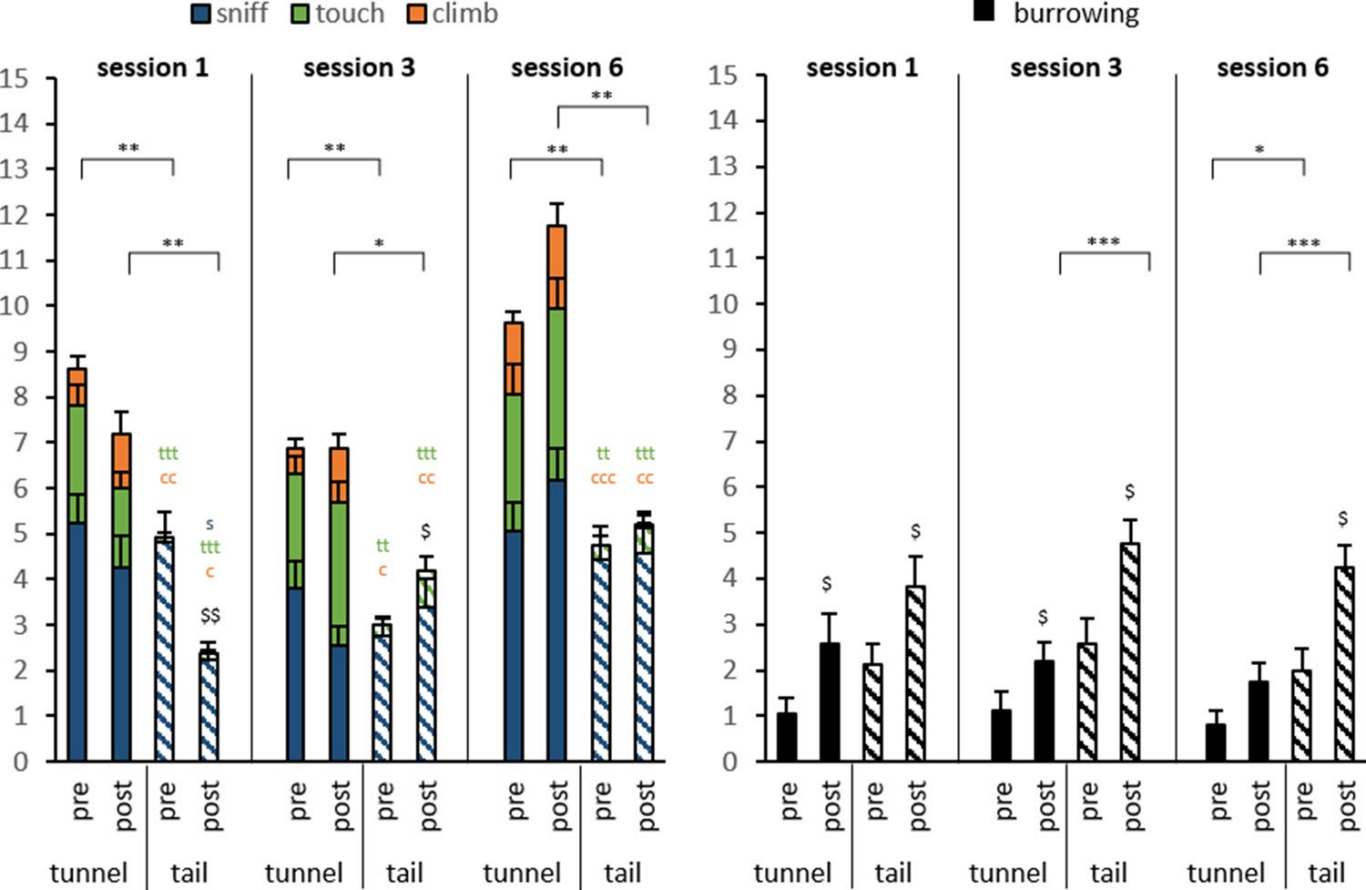
Voluntary interactions of daily handled mice with the handler before and after the handling session in the course of the initial handling. Left: explorative and positive behaviours (sniffing, touching, climbing). Right: defensive burrowing behaviour. N = 16, Data are represented as means ± SEM. *Differences of exploratory behaviours; s: sniffing approaches s < 0.05; t: touching approaches t < 0.05, tt < 0.01, ttt < 0.001; c: climbing approaches c < 0.05, cc < 0.01, ccc < 0.001; $: differences between pre and post handling.

During the first two weeks of the respective handling techniques, we conducted the *interaction test*. We evaluated exploratory behaviours both before and after the handling (Fig. 2).

In general, the tunnel handling led to more initial (= pre handling) positive interactions, such as touching (technique: F(1,90) = 60,399, p < 0.001) and climbing (technique: F(1,90) = 46.735, p < 0.001). Sex-specific differences were only observed in session 6 (sex: post F(1,28) = 8.958, p = 0.006) when females showed less approaches. In general, exploratory sniffing was not affected, but tail handled mice showed only a few touch responses and nearly no climbing. The defensive burrowing behaviour, on the other hand, was more pronounced in tail handled mice (technique: F(1,90 = 12,417, p < 0.001).We found differences between the test sessions for sniffing (session: F(2,90) = 4.627, p = 0.01, post hoc day 3 vs 6 p = 0.058) and climbing (session: F(2,90) = 4.401, p = 0.015, post hoc day 3 vs 6 p = 0.016). Both parameters increased comparing session 3 and 6.

The differences between the techniques was also prominent after the handling in the interaction test: touching (technique F(1,90) = 51.653, p < 0.001) and climbing (technique F(1,90) = 28.310, p < 0.001) was more prominent in tunnel handled mice. Interestingly, the measurement after the handling detected a difference between the techniques (F(1,90) = 22.672, p < 0.001). Sniffing remained unaltered. With respect to development of interaction over sessions, we detected a trend for increasing sniffing behavior (session: F(2,90) = 2.752, p = 0.069) and a significant effect for touching (session: F(2,90) = 5.258, p = 0.007, post hoc day 1 vs day 3 p = 0.037 and day 1 vs day 6 p = 0.010).

Differences in exploratory behaviour before and after handling were only detected after tail handling. In session 1 tail handling evoked reduced exploration (handling intervention: F(1,14) = 19.386 p = 0.001), which was reversed in session 3 (handling intervention: F(1,14) = 5.458 p = 0.035) and finally non-present in session 6. Defensive behaviour increased after the handling intervention in both handling techniques throughout the sessions—except for the tunnel handling in the final session.

## Discussion

Tail handling is considered aversive to rodents as it resembles the feeling of being caught by a predator^4^. The evolutionary instinct is to flee and avoid. Handling with a tunnel or other alternative handling techniques like cup handling do not provoke such an instinctive reaction^17^. We hypothesized that tunnel handling circumvents potential stress for laboratory mice, preventing negative effects on affective behaviour and thus refining experimental procedures. In this study, we focused on the initial adaptation to tunnel handling in the most widely used mouse strain in medical and behavioural research: C57BL/6. We found that both stress hormone release and affective behaviour were influenced by the handling technique and handling frequency in a sex-dependent manner.

Tunnel handling has already been validated as a tool to reduce anxiety-like behaviour in male and female mice^4,5^. Clarkson and Dwyer also demonstrated that tunnel handling led to less anhedonia-like behaviour compared to tail handling^3^. Our results corroborate the observation of a depressive-like phenotype of tail handled mice in the FST for males. The anhedonia study did not assess female mice, so their anhedonic status remains unknown. We did not detect despair-like behaviour in females. Further research is necessary to see, if possibly females are simply less sensitive to tail handling.

In males, tail handling triggered both anhedonic and despair behaviours which are typical characteristics of animal models of depression, e.g. stress-based models such as chronic mild stress (CMS)^22,39^. However, while CMS protocols often include a great variety of stressors, the daily tail handling was limited to one type of stressor and, unlike many of the CMS protocols^40–42^, the daily handling stress was predictable. Anxiety might play a role in the establishment of CMS-induced depression, but some stressors are known to be aversive without being anxiety-focused, e.g. tilted cages, changes in circadian rhythms by continuous illumination or heat stress^43^, while tail handling is known to induce anxiety^10^. Perhaps the permanent exposition to the anxiogenic handling is one explanation for the specific effect on males—as some studies showed how female rodents typically display less anxiety-like behaviour^44,45^. The stress load of handling could be too small to affect females on a behavioural level. Males however could have a lower threshold of reacting to this stimulus. Interestingly, other studies showed no differences in anxiety-like behaviour due to tail handling^5,8^. Therefore, the origin of the sex-dependent effect is hard to pin down with the current results. A direct comparison of daily handling with a non-anxiogenic stressor could be used to confirm this idea.

Apart from depression-like behaviour, tail handling also reduced the performance in burrowing in the home cage setting. This parameter is commonly used as a marker for rodents’ wellbeing^29^. However, the handling frequency influenced the outcome: wellbeing-associated parameters were only affected in those animals which were handled daily. This indicates that depressive-like behaviour does not necessarily lead to abnormal burrowing behaviour, i.e. reduced wellbeing. Therefore a burrowing mouse may have impairments in affective behaviours, but a mouse that does not burrow is more likely to display such impairments. Apparently, the affective state of a mouse cannot be detected by a single wellbeing parameter, but it can be still used as an indicator^16,29,46,47^. More research is necessary to illuminate the full connection between burrowing behaviour and the affective state^46^. Nonetheless, we found burrowing to be a sensitive tool for the assessment of effects in males induced by daily tail handling. In female mice, however, we did not detect any welfare or affective impairments.

FCM concentrations reflect adrenocortical activity and thus the stress hormone response, well^48^. Some studies have already observed a sex-dependent corticosterone increase, where females appeared to have higher corticosterone levels after stressful events^49,50^. Generally, FCM levels are higher in females due to differences in the metabolism^37,38,49,50^. Unfortunately, many studies solely used males, falsely assuming that the utilization of females always necessitates the observation of the oestrous cycle and inevitably increases the variability in results^51^. As we did include females in our study, we were able to observe higher FCM concentrations in weekly handled female mice. FCM concentrations of daily handled females and all males, however, appeared undisturbed. One explanation could be that the habituation to the low-frequency handling scheme was not as quick for females as it was for males and hence, this interaction protocol induced a stress hormone response. Repeated contact with the handler is known to habituate rodents to handling, when performed with procedures which are as stress-free as possible^5^ or follow an escalating protocol, in which mice are introduced to tail handling over several days starting with the simple presentation of the hand and short interactions. This introduction to the handling process led to a better initial performance in a cognitive test, possibly due to reduced stress and anxiety^52^. This observation is in line with the reduced FCM concentrations of the daily handled female mice in our study, underlining the benefit of habituation on the stress response. In general, females, irrespective of the handling method, showed higher concentrations of plasma corticosterone^53^, proposing that females may be more sensitive to handling as for other types of stressors^54,55^. The analysed faecal samples were collected 12 days after the onset of the respective handling, which might have been a sufficient time frame to adapt to the handling stress for all males and the daily handled females, but not for the weekly handled females. We think it is particularly noteworthy that, although the FCM concentrations of males were not influenced by handling technique or handling frequency, we confirm significant stress-associated affective and wellbeing associated impairments with an increase in the despair behaviour^56,57^ and a reduced burrowing in the home cage^29,58^, while females remained unaffected regarding behaviours in these domains. This supports the fact that in most murine models females exhibit less anxiety in behavioural tests than males^59^.

Both sexes demonstrated more interaction with the tunnel than with the hand. We further specified the interaction between the subject and the handling instrument into different categories, to analyse the behaviour more in detail. Sniffing was categorized as neutral exploration. In contrast, we classified touching and climbing onto the handling instrument as a positive interaction, demonstrating the willingness for voluntary physical exposition and signaling a feeling of safety. Lastly, we identified burrowing during the interaction test as a defensive behaviour, comparable to the one seen in the novelty-induced anxiety, in which this defensive burrowing behaviour takes place after the exposure to a novel yet harmless object^60,61^. While both, the exploration and the positive behaviours, were prominently enhanced towards the tunnel in comparison to the hand, defensive burrowing was more pronounced towards the experimenter’s hand, especially directly after the interaction. In line with other studies^5,7^, the hand appears more aversive to the animal. In summary, the tunnel was explored more often and the quality of approaches was more positive and less defensive^17,62^.

Our findings are limited to individually housed experimental mice. Mice are social animals and are preferably housed in groups^63–65^. There are however, situations in which individual housing can be appropriate, especially in male mice, where single housing avoids aggressiveness towards conspecifics—which is a welfare issue. Besides the obvious injuries and pain, the frequent exposure to social distress can lead to depression-like states in attacked lower ranked animals^66^. In female mice aggression is very rare and hence this argumentation does not hold up. But for comparability reasons between the groups they might be socially isolated as well. A systematic evaluation whether group-housed females might be a suitable control group to males is not available. In general, social isolation is considered a burden for female mice and from this perspective it is interesting to see, that the females in our study showed no alteration in behaviour, but only an elevated FCM response to weekly handling independent from the handling technique. Single housed males were sensitive to tail handling with respect to despair and burrowing. However, Mertens et al.^67^ showed that tail handling can still be an appropriate handling technique for male mice, as it reduces aggressiveness after the transfer to new cages in group-housed C57BL6/N. Since tail handling did not negatively influence the mice compared to tunnel handling—except for anxiety-related behaviour—they even propose tail handling as a refinement to prevent aggression, which promotes the beneficial group housing over social isolation.

The ethical principle of the 3R (replace, reduce, refine) is to minimize the number of animal experiments while maximizing the welfare of laboratory animals and the produced results^68^. Scientists have largely committed to it and it is now integrated into legislation in the EU directive 2010/63. Especially concerning refinement, handling techniques have been a matter of intense debate in recent years. The most common procedure, tail handling, has been shown to induce not only anxiety but also depression-like behaviour. Alternative techniques to circumvent these strains and stresses are valuable, but only sparsely implemented in daily routines nowadays. The feasibility of tunnel handling is still being discussed. Many researchers see the tunnel solely as a tool for environmental enrichment, which is implemented into the home cage. And while environmental enrichment is a valuable tool for increasing the welfare of animals^69^, it can also become a confounding factor in experiments^70^. Therefore, some researchers might balk at the idea of an additional factor to consider in their experimental design. However, it is not necessary to enrich the home cage in order to handle them with the tunnel. Gouveia and Hurst demonstrated that the use of a tunnel, which served as a permanent enrichment within the home cage of the animals, was initially preferred over a tunnel, to which the animals were only exposed to during handling^5^. But in the long run, both tunnel-based approaches showed similar responses in the elevated plus-maze, when compared to a tail handled group, signifying the higher impact of the handling technique over the familiarity of the handling instrument^5^. Moreover, the welfare effect of tunnel handling might be difficult to detect in wild type mice, while it might be easier in disease models. In a chronic kidney disease model, for instance, the severity of symptoms ameliorated in female mice^49^.

Some studies have found evidence for reduced experimental variation due to tunnel handling. The mean variation is one of the parameters that define the statistically necessary group size. Lower variation can lead to the reduction of animals needed to achieve meaningful scientific results^71^. Fridgeirsdottir and Hillered^52^ found that the gentle habituation of escalating handling led to reduced variation in the Morris water maze test. Nakamura and Suzuki^8^ found decreased variation in the open field and elevated O-maze test in tunnel handled compared to tail handled mice. In our study, we did not observe reduced variability in the results of our experiments. Further and repeated experiments would be necessary to fine-tune the method and to analyse whether handling can also be successfully used to reduce the number of animals, independent of the experimenter and lab. Another concern is that changing the handling technique can be an incisive process as it may complicate comparison to former results, by introducing this additional factor. On the other hand, environmental stressors as tail handling can reduce reliability and reproducibility^14,72^.

A conversion to tunnel handling as a standard operation procedure in also in large scaled facilities is accompanied with substantial alterations of processes with new steps of procedure, e.g. cleaning of tunnels. It can also raise costs for purchasing of new material, especially if the permanent home enrichment is put in place, and follow-up costs, e.g. by cleaning^5,17^. In addition, this new materials needs storage capacity. Hence, economic evaluations need to be considered to estimate whether the investment is worthwhile in each particular case. Various economic tools are available according to the objective of the analysis; cost–benefit analysis (CBA) and cost-effectiveness analysis (CEA) are the most relevant^73^. In fact, while CBA uses monetary terms for evaluating costs and outcomes to provide information about the financial feasibility of the project, CEA considers the relation between costs and effectiveness of two different interventions. Both can be useful to highlight the potential benefit of non-aversive handling in terms of allocation of resources and comparison with the state of the art techniques^74,75^. However, merely economic evaluations are not sufficient to justify the adoption or rejection of a new procedure, while animal wellbeing and experiment reproducibility should be the main drivers. Indeed, more research is necessary to understand how handling influences the phenotype to prevent misleading scientific results and feasibility evaluation of whether or not to use tunnels instead of tail handling should be assessed not only comprising the potentially initial higher workload and costs, but also focusing on the wellbeing of the subjects. Apart from the obvious ethical aspects, impaired wellbeing is a confounding factor for many readouts in animal experiments. Whenever the affective state of the experimental animals plays a role in the research question of a study, the usage of an alternative handling method (like tunnel handling) seems an advisable approach.
